# Estimating the Energy Expenditure of Grazing Farm Animals Based on Dynamic Body Acceleration

**DOI:** 10.3390/ani14152140

**Published:** 2024-07-23

**Authors:** Pedro Gonçalves, João Magalhães, Daniel Corujo

**Affiliations:** 1 Escola Superior de Tecnologia e Gestão de Águeda and Instituto de Telecomunicações, Universidade de Aveiro, 3810-198 Aveiro, Portugal; 2 Departamento de Eletrónica Telecomunicações and Informática and Instituto de Telecomunicações, Universidade de Aveiro, 3810-198 Aveiro, Portugal; jrsrm@ua.pt (J.M.); dcorujo@ua.pt (D.C.)

**Keywords:** energy expenditure, accelerometry, animal behavior

## Abstract

**Simple Summary:**

In this paper, we developed and assessed a method for measuring the energy expenditure of ruminants using data collected from wearable inertial sensors that monitor accelerations. The measured values were compared with reference ones for animal expended energy, as reported in the literature, to evaluate the quality of the measurement. A results comparison allowed us to verify that the obtained values were within the reported reference ranges, which is very promising as it is a specific method with a low impact on animals’ daily lives. Albeit not offering precision comparable to direct measurements, the measurement produced can be integrated with other complementary sources of information, (e.g., the evolution of the animal’s weight, ingestion time) and can thus provide extremely useful information for the animals’ feed management process.

**Abstract:**

Indirect methods of measuring the energy expenditure of grazing animals using heartbeat variation or accelerometers are very convenient due to their low cost and low intrusiveness, allowing animals to maintain their usual routine. In the case of accelerometers, it is possible to use them to measure activity, as well as to classify animal behavior, allowing their usage in other scenarios. Despite the obvious convenience of use, it is important to evaluate the measurement error and understand the validity of the measurement through a simplistic method. In this paper, data from accelerometers were used to classify behavior and measure animal activity, and an algorithm was developed to calculate the energy expended by sheep. The results of the energy expenditure calculations were subsequently compared with the values reported in the literature, and it was verified that the values obtained were within the reference ranges. Although it cannot be used as a real metering of energy expended, the method is promising, as it can be integrated with other complementary sources of information, such as the evolution of the animal’s weight and ingestion time, thus providing assistance in animals’ dietary management.

## 1. Introduction

Measuring the energy used by animals helps determine the necessary food intake for replenishing energy levels, directly influencing the cost of feed and, consequently, the financial outcome of livestock farming. On intensive or semi-intensive livestock farms, animals are commonly fed ad libitum once back in the shelter. An increase in the amount of food dispensed does not translate into a better result in activity, be it milk production or meat farming, nor in terms of animal welfare. Also, animals, when satiated, reject food, which cannot be reutilized due to the degradation associated with fermentation or the addition of bacteria, something that translates into inefficiency in livestock farming.

Examining animal activity [1], especially in pasture animals, is of primary importance because it impacts animals’ energy expenditure. The muscular work necessary to carry out activities, such as locomotion, demands energy, which has a huge impact on an animal’s daily energy expenditure [2].

Direct measurements of energy expenditure are difficult to perform, which is why several proxy methods [3] have been developed over time, which indirectly infer energy consumption. Examples are the measurement of the volume of oxygen (VO_2_) [4], usually within respiration chambers [2]; the measurement of dynamic body acceleration [5]; or the measurement of heart rate [6].

The methods based on the measurement of VO_2_ typically consist of placing animals inside breathing chambers, using treadmills to simulate the conditions of animal life [5]. This is a costly method, both for the study and for the animal, and not suitable for analyzing energy expenditure during their free-ranging activity. Despite the convenience of the method and the possibility of monitoring animals in a free regime, measuring energy through heart rate presents serious calibration challenges.

Like heart rate monitors, inertial sensors are convenient and inexpensive and allow for the free-range monitoring of animals, either in a barn or in pasture, and do not present the same calibration problems. Additionally, they have the merit of being able to be used to classify animal behavior [7], such as detecting feeding behavior, something of great importance for scenarios related to the optimization of animal energy balance. Inertial sensors have been successfully used in different methods to infer the amount of animal activity expenditure, such as measuring the number of steps taken [8,9], or through dynamic body acceleration (DBA) [5], which is considered a method with lower error [10] and which shows a greater correlation with measures based on heartbeat count [6].

This paper presents an implementation of an energy expenditure indicator for grazing sheep, based on DBA, which does not allow the absolute calculation of the energy expended but which, together with other sources of information, can provide valuable information about animals’ food needs. It is organized as follows. Section 2 analyzes related work on proxy methods for energy expenditure based on inertial sensors and Section 3 presents the energy expenditure mechanism. Section 4 presents the mechanism assessment results and Section 5 discusses the obtained results and compares them with the ones reported in the literature. Finally, Section 6 concludes the paper, enumerating major conclusions and defining future lines of work.

## 2. Related Work on Proxy Methods for Energy Expenditure

Animal energy expenditure depends on intrinsic factors such as their weight, well-being, and sexual life or on extrinsic factors such as temperature, location, and whether they are stabled or free-ranging.

The dynamic behavior of an animal, for example, has a huge impact on the energy spent, given the energy required by muscular activity, and there are huge differences depending on the dynamism of the behavior itself. Locomotion requires a much higher amount of energy than static behaviors, and this cost increases with the movement’s speed [5,11]. Regarding this, there are reported estimates of a 2.5-fold increase in energy expenditure between walking and running behavior [12]. In addition to the speed of locomotion, the relief of the terrain and the surface substrate also impact the energy spent in animal locomotion. When animals move uphill, they must expend energy to overcome gravitational force, a value that depends on their weight. But, when they descend, despite the movement being favored by gravity, they also expend energy to control their descent [2,13]. Likewise, the surface substrate has a considerable impact on animal locomotion due to the force absorption associated with locomotion on the ground, an effect that has been reported in the literature for both animals [9] and humans [14].

Temperature is also a factor with a considerable impact on the energy expenditure of sheep since, like other mammals, they have thermoregulation mechanisms that require energy. They present a comfort zone between 22 °C and 30 °C [5], where the change in ambient temperature has no effect on the energy expended, but if below or above this range, the values increase. At colder temperatures, the increase in energy expenditure is explained by the energy cost to maintain their body temperature to cope with the heat loss due to the environment. At higher temperatures, animals must spend energy to cool down [15]. Likewise, the removal of wool has a non-negligible effect, as shown by Blaxter et al. [16], where sheep with fleeces have very wide thermoneutral zones, in contrast to closely clipped sheep.

### 2.1. Energy Based on Activity

Proxy methods [3] for determining energy expenditure based on DBA have, over time, used different ways of obtaining dynamic acceleration: first, overall dynamic body acceleration (ODBA), which sums the dynamic acceleration from three orthogonally placed accelerometers that represent the main axes of an animal’s body, and second, vectorial dynamic body acceleration (VeDBA). Both are mathematically represented in Equations (1) and (2).

(1) $$ODBA=\left|{Acc}_{x}\right|+\left|{Acc}_{y}\right|+\left|{Acc}_{z}\right|$$

(2) $$VeDBA=\sqrt{{Acc}_{x}^{2}+{Acc}_{y}^{2}+{Acc}_{z}^{2}}$$

Qasem et al. [10] used several species in a study comparing ODBA and VeDBA to determine the better proxy for oxygen consumption rate and establish the better proxy for energy consumption. Several species were used, including humans, and data were collected while moving at different speeds on a treadmill. The results confirmed both ODBA and VeDBA as valid proxies for energy consumption, but they proved that ODBA requires the orientation of the accelerometers to be consistent between individuals. For arbitrary orientations of the accelerometer, ODBA can both overestimate and underestimate changes in the acceleration magnitude, something already reported by Spivey et al [17].

Direct animal energy expenditure measurements are often carried out by measuring the VO_2_ consumed by animals during activities [4], which is why the mathematical expressions that define energy expenditure are expressed as a function of VO_2_.

The mathematical relationship between VO_2_ and the energy consumed by metabolism mechanisms is based on the amount of energy released during the aerobic metabolism of energy substrates such as carbohydrates, fats, and proteins. The relationship between oxygen consumption and energy production can be expressed through the concept of respiratory equivalent (RE), which is the amount of oxygen consumed to produce a unit of energy, generally expressed in liters of oxygen per calorie.

(3) $$RE=\frac{VO_{2}}{Energy}$$

The respiratory equivalent may vary depending on the metabolized substrates, with values between 5 L O_2_/cal for proteins, 4.7 L O_2_/cal for fats, and 4.3 L O_2_/cal for proteins.

The relationship between the volume of oxygen consumed and body acceleration has been the subject of extensive work with a huge diversity of animal species. Mulvenna et al. [18] conducted a study with domestic sheep (*Ovis aries*) to evaluate the ability of VeDBA obtained from tri-axial accelerometer data loggers to act as a proxy for energy expenditure in non-uniform environments. Animal oxygen consumption was measured at different velocities and inclinations over a treadmill. According to their results, an immobile animal consumes the following:

(4) $$VO_{2}=3.67\;\frac{mL\;O_{2}}{kg\;min}$$

A walking animal on a 0-degree plane consumes the following:

(5) $$VO_{2}=13.72\;\times \;VeDBA\;\left(g\right)+3.67\;\frac{mL\;O_{2}}{kg\;min}$$

A walking animal on an incline consumes the following:

(6) $$VO_{2}=36.31\;VeDBA\;\left(g\right)+3.67\;\frac{mL\;O_{2}}{kg\;min}$$

Dickinson et al. [5] studied the interactions between behavior, energy expenditure, ambient temperature, and terrain slope, measuring the rate of VO_2_ in pygmy goats (*Capra hircus aegarus*) using an open-flow indirect calorimetry. They tested the consumption of VO_2_ in temperatures between 9.7 and 31.5 °C during resting behavior, at walking speeds between 0.8 and 3.0 km h^−1^, and on different inclines (0, +15°, −15°). Their results confirmed both the thermal regulation impact and the DBA impact on energy expenditure, and obtained energy expressions for different behaviors, shown in Table 1. 

Considering that typical RE values are between 4.3 and 5, as this value depends on food composition, which, in pasture, is mostly composed of proteins, we can consider a final RE value near 5. Converting the VO_2_ expressions from (4), (5), and (6), we obtain similar expressions for each of the behaviors from Table 1.

### 2.2. Behaviour Identification

The identification of animal behavior based on wearable inertial sensors [7] has long been successfully carried out using accelerometers and gyroscopes applied to collars [7], harnesses [20], or earrings [21]. Work reported in the literature has considered hectograms related to the control of animal welfare [22] and production, or even for purposes related to animal conditioning [23].

Gathered sensor data are often used by machine learning algorithms, which, after building a learning model, classify the data according to the learned model. Fonseca et al. [7] developed a collar-mounted inertial sensor envisaged to monitor sheep behavior. Its hectogram included feed-related behavior, and the collar integration with the gateway allowed for real-time monitoring. Therein, activity data were enriched with external data, such as data relating to the animal’s weight and weather data, improving estimates regarding the energy expended by each of the animals.

## 3. Materials and Methods

In this paper, animal monitoring data were gathered through wearable inertial sensors and used as an indirect way of inferring the energy expended by animals. To achieve this, accelerometry data from grazing sheep were gathered and supplemented with weather information taken from the national weather forecast entity. The animal behaviors were then classified using an ethogram designed to optimize the analysis of the animals’ dynamic behavior, and the animals’ activity was measured using raw accelerometry data. Finally, the energy expenditure values were calculated according to the procedure described below.

### 3.1. Data Gathering and Preparation

The activity data were collected by a collar manufactured by iFarmTec (Gafanha da Encarnação, Portugal) [24], which was placed around the sheep’s necks, with the animals maintaining their daily habits during two periods, namely, from September to December 2022 and from January to March 2023. The monitoring system included a gateway that implemented an opportunistic communications system based on Bluetooth Low Energy [25] that allowed for the transfer of information between the collar as soon as it was within radio range of the gateway placed in the shelter. During the day, the collars stored the data collected by the sensors of the animals grazing in the meadow for later transfer. Once the animals arrived at the shelter, the collars established a connection with the gateway and transferred the monitoring data stored during the grazing period.

For the present work, data from a single day, 24 h, of two animals were used: one sheep that remained on free pasture, which we call the active animal, and a second sheep kept in a barn, which we designate as the inactive animal. After being collected from the gateway, the data were cleaned, with damaged records and outliers being removed.

The data were then processed to remove the contribution of gravity from the collected accelerations and obtain the accelerations due to the animal’s movement. The strategy followed to obtain the acceleration of each of the axes consisted of obtaining the differential acceleration of that axis in relation to the previous sample, as defined in Equation (7).

(7) $$\left\{\begin{array}{c} A_{x}=A_{x}-A_{x-1}\\ A_{y}=A_{y}-A_{y-1}\\ A_{z}=A_{z}-A_{z-1} \end{array}\right\}$$

**Figure 1:**
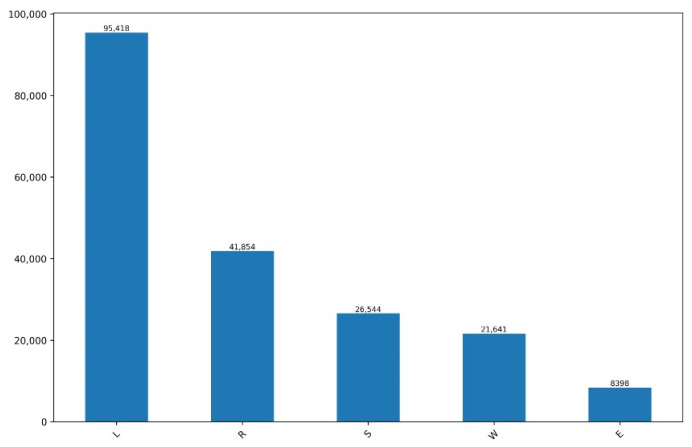
Count of occurrences of each behavior.

For the purposes of behavior classification, a classification algorithm was created using a machine learning technique, considering using the behaviors of Eating, Lying down, Ruminating, Standing, and Walking, according to the procedure described in [7]. The process included the monitoring and annotation of the accelerometer data, based on previously recorded video images of animals wearing the collars while they remained free-grazing. The accelerometry data were then taken from the monitoring system and supplemented with using video recordings. Figure 1 shows the distribution of the annotated accelerometry data, which contained 193,546 records, distributed by each of the behaviors of the defined ethogram, after the supervision process.

The supervised data were subsequently subjected to a machine learning process using an algorithm based on decision trees, which classified the behaviors included in the desired ethogram. Finally, weather information was provided by the Portuguese Institute of Sea and Atmosphere [26], from the nearest meteorological station, and integrated with the accelerometry data.

### 3.2. Energy Measurement

**Figure 2:**
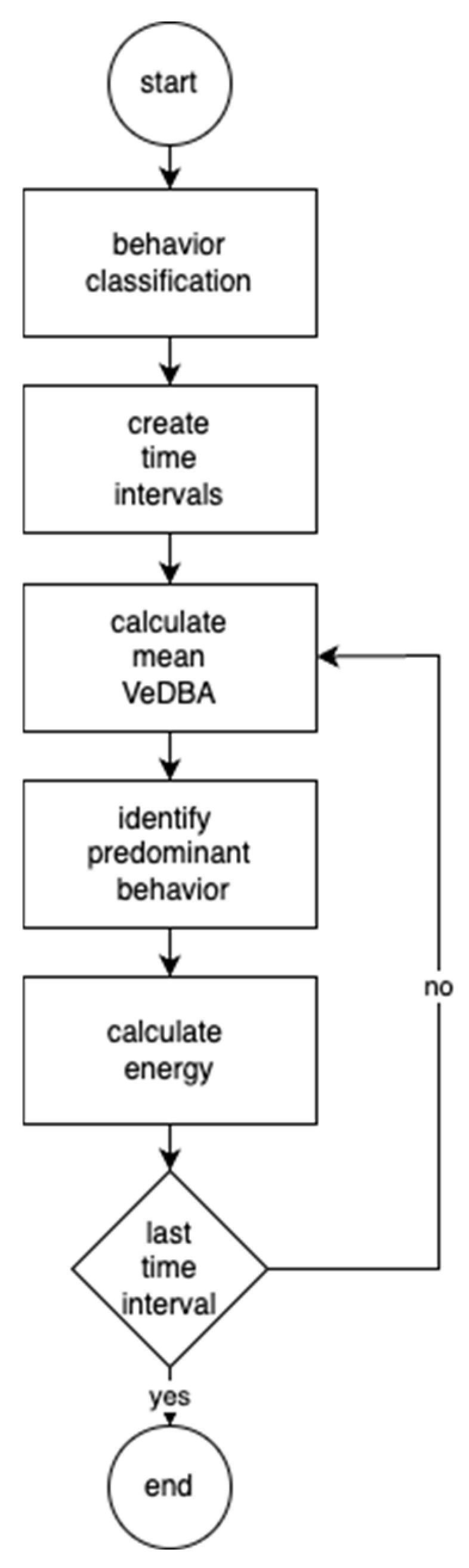
Energy calculation process.

The calculation of energy expended followed the methodology defined in [5], and the calculation procedure is illustrated in Figure 2. The procedure began by classifying the behavior of each monitoring record using the decision tree algorithm, aggregating the monitoring data into temporal intervals, and determining the energy expended in each interval.

Monitoring data aggregation consisted of dividing the monitoring time into one-minute intervals, and, for each of them, (i) calculating the mean VeDBA for the interval, (ii) identifying the predominant behavior in the interval, and, (iii) finally, calculating the energy associated with the predominant behavior, taking into account the determined value of VeDBA calculated for the interval and the temperature, according to the energy values defined in Table 2. Finally, a weight of 60 kg was also stipulated for the animals.

Being an indicator of energy expenditure, with no intention of carrying out exact measurements, some simplifications were made in the energy calculation process. Firstly, the impact of movement speed on energy expenditure was disregarded because running behavior was not included in the ethogram. And assumptions were made about slope of the land, floor type, and food composition. Also, in relation to the speed of movement, a simplification was made, considering that running behavior would be less common for pasture animals, so, whenever there was movement, the energy calculation was made based on the energy consumption associated with the only state related to locomotion considered in the present study, which was walking behavior. Moreover, a weight of 60 kg was considered; it was assumed that the pastureland was flat, with no relief. Thirdly, the effect of the type of soil was ignored, ignoring the friction related to the movement of animals over the grass, and meteorological information data provided by the Portuguese Institute of Sea and Atmosphere [26] from the nearest meteorological station. Finally, they were not considered any aspects related to animal feeding over the energy expenditure.

## 4. Results

**Figure 3:**
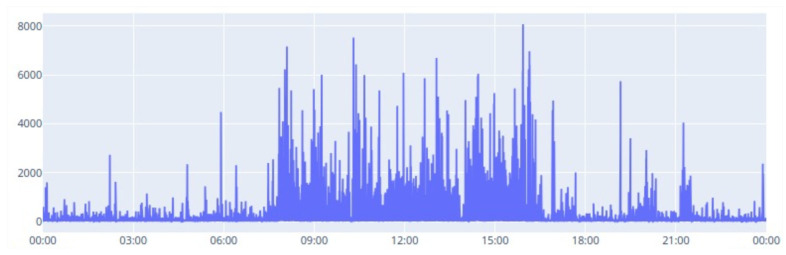
Daily activity evolution.

The activity analysis, plotted in Figure 3, demonstrated activity with variations throughout the day, with significantly higher values during the daytime period compared to the nighttime period. During the night, accelerations presented smaller variations, with peaks below 3000 mG, while, during the day, the variations were more frequent and with peaks with much greater amplitudes, with values reaching 8000 mG.

The model, as shown in Table 3, demonstrated relatively robust performance, with an accuracy of 86%. Behavior L presented the best performance result in terms of precision, recall, and F1-Score, while behavior E presented the lowest values. The weighted average indicated that the model was balanced in classifying the different classes. A closer examination revealed that the model’s performance deteriorated as the number of samples used to train it decreased due to the limited size of the dataset.

**Figure 4:**
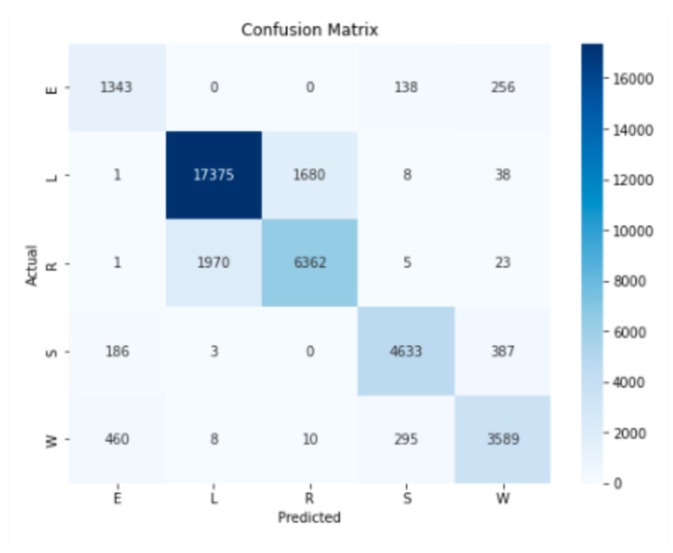
Confusion matrix for the learning process.

The confusion matrix analysis (Figure 4) allowed us to confirm the previously analyzed results and verify the confusion between the L and R states. There was an additional reason for this confusion. Both animal behaviors were characterized by very sedentary behavior as the animals performed much of their rumination lying down, as in behavior L.

**Figure 5:**
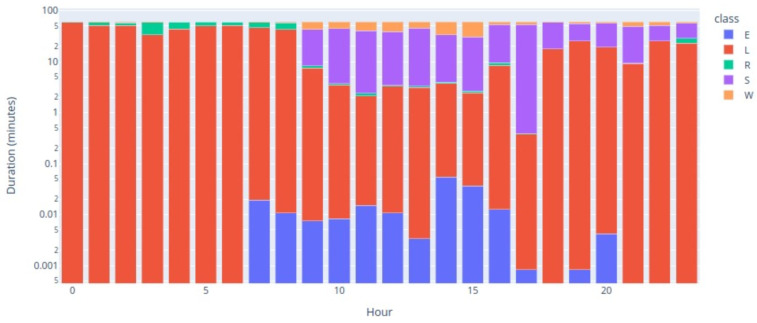
Echotomogram behaviors over the day.

Figure 5 shows the hourly distribution of each behavior throughout the day, a classification that was carried out using the RF algorithm with the metrics also presented previously and represented in Table 3.

Analyzing the distribution of behaviors, through Figure 5, we can see that behaviors L and R were predominant during the night hours, although some records of R may have been confusion between the two by the model. Furthermore, at later stages of the day, there were many L records, which may also consist of misidentified R behavior. Behavior W had a sharp increase in occurrences between 9 am and 3 pm, which was perfectly normal since the data corresponded to the monitoring of an animal on pasture during that period. Behavior S began to show greater frequency also from 9 am and, during the day, it remained frequent until the end of the day. Behavior E had sporadic occurrences distributed throughout the region, which is in line with the feeding behavior on pasture and with the confusion that was made between behaviors W and S, meaning that some occurrences should have been counted as behavior E.

**Figure 6:**
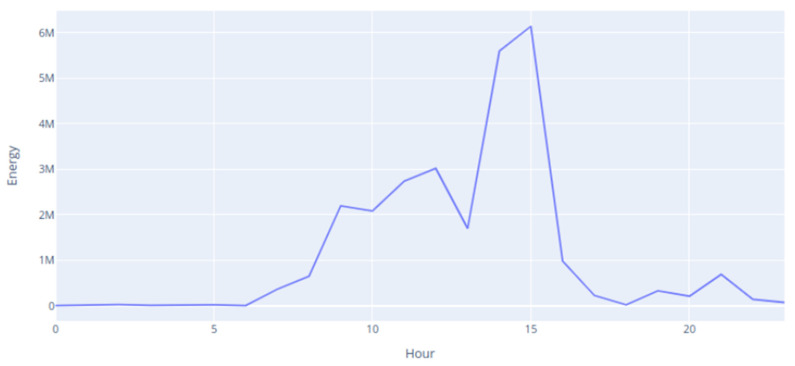
Energy expended during the day.

The energy expended by the animals throughout the day, calculated based on the behaviors that they performed during their daily life, using the methodology defined in Table 2, is represented in Figure 6.

The sheep whose energy expenditure is represented in Figure 6 was freely roaming during the hours of the day (between 9 am and 4 pm). During the night, until 6 am on that day, there was very low energy expenditure. After dawn, the energy expenditure increased a lot and remained at high values until the time of collecting the animal from the shelter. Also, in the following hours, the energy spent was much lower than the energy spent during the day, but more energy was spent during the early hours of the morning.

**Figure 7:**
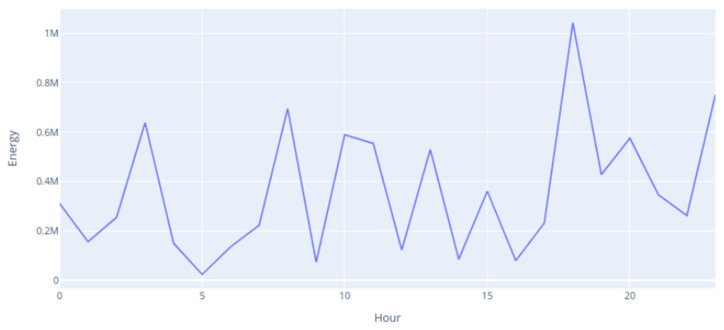
Inactive animal energy expenditure during the day.

To evaluate the results obtained, data from two animals with very different behaviors were used. One of the animals maintained its daily behavior, spending the day grazing in the meadow, whereas the other was kept in a shelter throughout the monitoring period. Figure 7 represents a graph of the energy spent by the sheep kept in the shelter. In this case, the energy spent throughout the day fluctuated, with several peaks associated with periodic movements, and values never coming close to the values expended by the active sheep.

**Figure 8:**
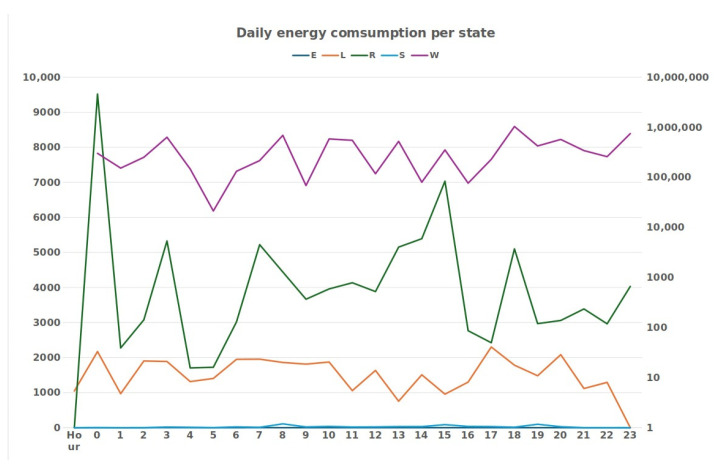
Active sheep energy expended per behavior.

Table 4 summarizes an hour-by-hour accounting of times spent by active animals in each state. As can be seen, during the night, the sheep was practically always lying down or chewing the cud. During the hours when it was free-ranging, there was a change in behavior that led to the behaviors of walking and standing being the most frequent. By the time the sheep returned to the shelter, behavior L increased and behavior W decreased. Figure 8 shows the value of energy spent by each behavior throughout the day for the active sheep. The graph shows that most of the energy was spent on walking, which is understandable, given the impact of this behavior on energy expenditure. The eating behavior had little energy consumption because this behavior was predicted a few times due to the confusion that existed in the model between this behavior with the behaviors of standing and walking.

**Figure 9:**
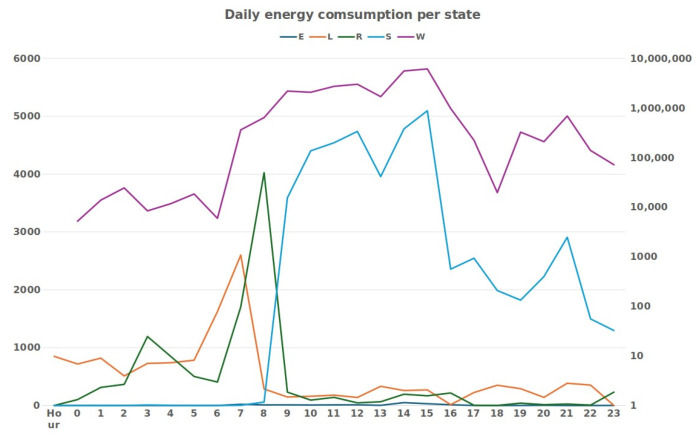
Inactive sheep energy expended per behavior.

Table 4 shows the time accumulated hour-by-hour for each behavior spent by the inactive ewe during the monitoring day. Its analysis allowed us to verify that the behaviors that were most adopted by the sheep when it was enclosed in a small space were L and R behaviors. The E behavior appeared with a very low frequency, something that can be explained by the inactivity and consequent lack of need for food or due to the classification error of the RF algorithm. In Figure 8 and Figure 9, the value of energy spent by each behavior throughout the day is graphically represented. The graph helps to understand that most of the energy was spent on behavior W, given the greater energy expenditure of this behavior.

Table 5 represents the daily energy cost of each behavior for both sheep. In the case of the active sheep, we can see that behavior E was the one that used the least energy, followed by R and L, with almost the same values. Behavior S was the second one that used more energy, but still in the same order of magnitude as the previous ones. Finally, the behavior that spent the most energy was W, which was orders of magnitude higher, as it is a behavior that requires more effort by the animal. In total, throughout the day, the active sheep spent 27,262.48 KJ.

Regarding the inactive sheep, we can see that behavior E was the one that used the least energy, following behavior S, a behavior rarely identified in the case of this sheep. This was followed by behavior L, despite being the one with the longest time identified and with the lowest energy cost. After this behavior, behavior R appeared. The behavior that used the most energy was W, despite only being identified for a short time, as it required more effort from the animal, as well as possibly more sudden movements, which involved the greatest expenditure of energy throughout the day. In total, throughout the day, the inactive sheep spent 8622.5 KJ.

## 5. Discussion

Animal activity, especially locomotive activity, has a massive impact on energy expenditure, given the need to expend energy in the production of work associated with movement. The analysis of activity throughout the monitoring day demonstrated a significant difference between nighttime activity and daytime activity in terms of frequency amplitude. During the night, the average amplitude was lower, although there were activity peaks related to the rest cycles typical of the night period [1]. During the day, the range of activity was considerably greater, despite being more irregular.

The results obtained in the learning process are of somewhat modest quality when compared to other works [23] and are due, on the one hand, to the fact that few samples were used in the process of building the classification model. On the other hand, and considering the confusion matrix, there was considerable confusion between some states, which also affected the final performance of the model: states L and R showed great similarities since, in both states, the animals were lying down for a large part of the time; states E, S, and W presented great confusion, given the roaming behavior of ruminants, which mediate the various behaviors during grazing, making recording difficult and translating into model classification errors.

The evolution of behavior throughout the day presents results consistent with previous studies [23] and demonstrates typical behavior during sheep grazing. During the night, the dominant behavior was L, alternating with periods of R. After the grazing period began, the animals alternated between states E, W, and S. Contrary to what was reported in [23], state L was not frequent, mainly occurring in the first hours of grazing while the animals were not satiated. This difference must be due to errors in classifying the behaviors previously mentioned.

The evolution of energy spent throughout the day followed the distribution pattern of behavior W due to the preponderant effect that this behavior has on global energy expenditure. To verify the validity of the results of this work, we analyzed daily animal monitoring data and compared the results obtained from an active animal, which spent part of the day grazing during birth, with data from an animal kept in a shelter. The results obtained through the analysis of data from the active animal showed much higher energy expenditure values, especially during the day; the animal kept in the shelter presented much lower energy expenditure values, distributed throughout the day.

The comparison between the frequency of behaviors and the energy spent on each behavior throughout the day demonstrates the dominant impact that behavior W had on energy expenditure and can help to make decisions about the quantity and time of supply of supplementary feed, both for animals in intensive and for animals in semi-intensive regimes.

The calculated total daily energy expended by the free-ranging animal totaled approximately 27.3 MJ, while, in the case of the animal kept in the shelter, it totaled 8.6 MJ. These values are in line with the indicative values referred to in the literature [27,28] since, considering the considered weight of the animal of 60 kg, they indicate values between up to 21.6 MK [27] and 29.2 MJ [28].

## 6. Conclusions

Daily energy expenditure has the greatest impact on an animal’s energy balance and, consequently, on their well-being and the food necessary to replace energy expended. The calculation of this expenditure plays a preponderant role in food adequacy and, consequently, in the optimization of livestock activities. The results obtained proved the enormous impact of more dynamic behaviors on the total energy expended and confirmed the feasibility of using inertial data to indirectly infer the energy expended by an animal.

The objective of the present work was to develop an indicator of energy expenditure based on animal activity, without the intention to establish an exact measure, given the limitations inherent in the use of an indirect method. Moreover, some well-known impacting factors on energy expenditure were not considered, such as the relief and type of soil, the velocity of the movement, and the animal feed composition.

In future work, tests with various elements of a herd, over several days, should be carried out to verify the quality of the results obtained. Additionally, the behavior supervision process should be improved to increase the behavior classification algorithm performance and consider the effect of terrain relief. Additionally, energy expenditure data should be added to data relating to weight evolution and feeding monitoring to allow livestock managers to make informed decisions regarding individual supplementation for each animal.

## Figures and Tables

**Table 1:** Description of the energetic costs of each behavior, the acceleration range observed for each behavior, and the line equations used to calculate the energetic costs of each behavior, adapted from [5].

Behavior	Description of Energetic Cost	Equation
Standing	VO_2_ while resting, measured in this study at different temperatures and accounting for DBA	$E=66.98+0.15\;T_{a}^{2}-7.97T_{a}+W+VeDBA$
Resting	Lying down, measured as using 29% less energy than standing for goats [19]	$E=\left(66.98+0.15\;T_{a}^{2}-7.97T_{a}+W+VeDBA\right)\;\times \;0.29\;$
Eating	VO_2_ while resting, measured in this study at equivalent DBA	$E=66.98+0.15\;T_{a}^{2}-7.97T_{a}+W+VeDBA$
Walking	Walking VO_2_, measured in this study at different speeds and terrain slopes at equivalent DBA	$E=-75.622+\alpha_{j}+\left(642.345+\gamma_{i}\right)\;\times \;VeDBA+T_{a}+W$
Running	The additional cost of this gait was estimated to be 2.5 times the energetic cost of walking [12]	$E=\left(-75.622+\alpha_{j}+\left(642.345+\gamma_{i}\right)\;\times \;VeDBA+T_{a}+W\right)\;\times \;2.5$

$T_{a}$ = ambient temperature, *W* = body weight, $\alpha_{j}$ = terrain slope ($\alpha_{l}$ = level, $\alpha_{p}$ = positive, $\alpha_{n}$ = negative), $\gamma_{i}$ = terrain slope ($\gamma_{l}$ = level, $\gamma_{p}$ = positive, $\gamma_{n}$ = negative).

**Table 2:** Echogram of process behaviors.

Behavior	Name	Description	Equation
E	Eating	Animal is eating	$E=66.98+0.15\;T_{a}^{2}-7.97T_{a}+W+DBA$
L	Lying down	Animal is resting, lying down	$E=\left(66.98+0.15\;T_{a}^{2}-7.97T_{a}+W+DBA\right)\times 0.29$
R	Ruminating	Animal is ruminating	$E=66.98+0.15\;T_{a}^{2}-7.97T_{a}+W+DBA$
S	Standing	Animal is resting, standing up	$E=66.98+0.15\;T_{a}^{2}-7.97T_{a}+W+DBA$
W	Walking	Animal is walking	$E=-75.622+\left(642.345\right)\times DBA+T_{a}+W$

**Table 3:** Model performance.

	Precision	Recall	F1-Score	Support
S	0.91	0.89	0.90	1737
W	0.84	0.82	0.83	19,102
E	0.67	0.72	0.72	8361
L	0.90	0.91	0.90	5209
R	0.79	0.76	0.78	4362
Weigh. Average	0.86	0.86	0.83	38,771

**Table 4:** Accumulated time per behavior.

	Active Sheep					Inactive Sheep				
Hour	E	L	R	S	W	E	L	R	S	W
0	0.00	58.93	1.06	0.00	0.01	0.00	21.15	38.80	0.01	0.05
1	0.00	51.53	7.36	0.00	1.18	0.00	44.88	15.08	0.00	0.02
2	0.00	51.33	7.08	0.01	1.59	0.00	30.78	29.18	0.00	0.02
3	0.00	33.87	25.76	0.03	0.34	0.00	32.00	27.79	0.11	0.13
4	0.00	43.95	15.96	0.02	0.07	0.00	48.29	11.56	0.03	0.10
5	0.00	50.05	9.79	0.00	0.17	0.00	47.04	12.90	0.01	0.07
6	0.00	51.45	8.53	0.00	0.02	0.00	38.81	20.83	0.13	0.23
7	0.02	46.71	12.58	0.00	0.69	0.00	35.46	24.14	0.02	0.37
8	0.01	43.33	14.92	0.39	1.35	0.00	37.70	21.00	0.38	0.91
9	0.01	7.52	0.73	35.06	16.69	0.00	41.71	17.92	0.07	0.30
10	0.01	3.51	0.23	41.11	15.15	0.00	34.19	25.44	0.13	0.24
11	0.02	2.12	0.28	38.29	19.30	0.00	34.75	24.99	0.07	0.20
12	0.01	3.27	0.16	35.47	21.09	0.00	30.76	28.95	0.08	0.20
13	0.00	3.11	0.22	42.46	14.21	0.00	31.54	28.02	0.15	0.28
14	0.05	3.66	0.23	29.56	26.48	0.00	22.32	37.28	0.14	0.23
15	0.04	2.41	0.19	28.20	29.18	0.00	28.04	31.70	0.15	0.14
16	0.01	8.39	1.23	44.38	5.99	0.00	34.46	25.10	0.18	0.24
17	0.00	0.39	0.01	52.69	6.92	0.00	41.81	17.84	0.13	0.24
18	0.00	17.91	0.00	41.69	0.39	0.00	33.65	26.16	0.06	0.12
19	0.00	25.52	0.04	30.48	3.96	0.00	33.12	25.82	0.38	0.68
20	0.00	19.41	0.03	37.95	2.61	0.00	38.51	20.95	0.22	0.31
21	0.00	9.13	0.16	40.65	10.07	0.00	37.54	22.38	0.00	0.04
22	0.00	25.34	0.06	26.22	8.38	0.00	28.44	31.52	0.00	0.08
23	0.00	23.15	5.37	28.34	3.14	0.00	19.16	39.34	0.00	0.03

**Table 5:** Daily energy expenditure for active animal vs. inactive animal.

Class	Energy Spent by Active Animal	Energy Spent by Inactive Animal Energy
E	0.17 KJ	4.2 J
L	12.89 KJ	37.1 KJ
R	10.92 KJ	96.2 KJ
S	47.82 KJ	0.63 KJ
W	27,155.5 KJ	8488.4 KJ
Total	27,260.3 KJ	8622.5 KJ

## Data Availability

Dataset available upon request from the authors.

## References

[1] Gonçalves P., Antunes M., Xavier W., Monteiro A. (2022). Flock Nocturnal Activity: Is There a Rotative Guard?. Appl. Sci..

[2] Clapperton J.L. (1964). The Energy Metabolism of Sheep Walking on the Level and on Gradients. Br. J. Nutr..

[3] Halsey L.G., Bryce C.M. (2021). Proxy Problems: Why a Calibration Is Essential for Interpreting Quantified Changes in Energy Expenditure from Biologging Data. Funct. Ecol..

[4] Halsey L.G., Shepard E.L.C., Quintana F., Gomez Laich A., Green J.A., Wilson R.P. (2009). The Relationship between Oxygen Consumption and Body Acceleration in a Range of Species. Comp. Biochem. Physiol.-A Mol. Integr. Physiol..

[5] Dickinson E.R., Stephens P.A., Marks N.J., Wilson R.P., Scantlebury D.M. (2021). Behaviour, Temperature and Terrain Slope Impact Estimates of Energy Expenditure Using Oxygen and Dynamic Body Acceleration. Anim. Biotelemetry.

[6] Miwa M., Oishi K., Nakagawa Y., Maeno H., Anzai H., Kumagai H., Okano K., Tobioka H., Hirooka H. (2015). Application of Overall Dynamic Body Acceleration as a Proxy for Estimating the Energy Expenditure of Grazing Farm Animals: Relationship with Heart Rate. PLoS ONE.

[7] Fonseca L., Corujo D., Xavier W., Gonçalves P. (2022). On the Development of a Wearable Animal Monitor. Animals.

[8] Shibata M., Mukai A., Kume S. (1981). Estimation of Energy Expenditure in Dairy Heifers Walking on the Level and on Gradients. Bull. Kyushu Agric. Exp. Station..

[9] White R.G., Yousef M.K. (1978). Energy Expenditure in Reindeer Walking on Roads and on Tundra. Can. J. Zool..

[10] Qasem L., Cardew A., Wilson A., Griffiths I., Halsey L.G., Shepard E.L.C., Gleiss A.C., Wilson R. (2012). Tri-Axial Dynamic Acceleration as a Proxy for Animal Energy Expenditure; Should We Be Summing Values or Calculating the Vector?. PLoS ONE.

[11] Maloiy G.M.O., Rugangazi B.M., Rowe M.F. (2009). Energy Expenditure during Level Locomotion in Large Desert Ungulates: The One-humped Camel and the Domestic Donkey. J. Zool..

[12] Parker K.L., Robbins C.T., Hanley T.A. (1984). Energy Expenditures for Locomotion by Mule Deer and Elk. J. Wildl. Manag..

[13] Halsey L.G., White C.R. (2016). A Different Angle: Comparative Analyses of Whole-Animal Transport Costs Running Uphill. J. Exp. Biol..

[14] Davies S.E.H., Mackinnon S.N. (2006). The Energetics of Walking on Sand and Grass at Various Speeds. Ergonomics.

[15] Speakman J.R., Król E. (2010). Maximal Heat Dissipation Capacity and Hyperthermia Risk: Neglected Key Factors in the Ecology of Endotherms. J. Anim. Ecol..

[16] Blaxter K.L., Graham N.M., Wainman F.W. (1959). Environmental Temperature, Energy Metabolism and Heat Regulation in Sheep. III. The Metabolism and Thermal Exchanges of Sheep with Fleeces. J. Agric. Sci..

[17] Spivey R.J., Bishop C.M. (2013). Interpretation of Body-Mounted Accelerometry in Flying Animals and Estimation of Biomechanical Power. J. R. Soc. Interface.

[18] Mulvenna C.C., Marks N.J., Wilson R.P., Halsey L.G., Scantlebury D.M. (2022). Can Metrics of Acceleration Provide Accurate Estimates of Energy Costs of Locomotion on Uneven Terrain? Using Domestic Sheep (*Ovis aries*) as an Example. Anim. Biotelemetry.

[19] Dailey T.V., Hobbs N.T. (1989). Travel in Alpine Terrain: Energy Expenditures for Locomotion by Mountain Goats and Bighorn Sheep. Can. J. Zool..

[20] Ikurior S.J., Marquetoux N., Leu S.T., Corner-Thomas R.A., Scott I., Pomroy W.E. (2021). What Are Sheep Doing? Tri-Axial Accelerometer Sensor Data Identify the Diel Activity Pattern of Ewe Lambs on Pasture. Sensors.

[21] Fogarty E.S., Swain D.L., Cronin G.M., Moraes L.E., Trotter M. (2020). Behaviour Classification of Extensively Grazed Sheep Using Machine Learning. Comput. Electron. Agric..

[22] Fogarty E.S., Swain D.L., Cronin G.M., Moraes L.E., Trotter M. (2020). Can Accelerometer Ear Tags Identify Behavioural Changes in Sheep Associated with Parturition?. Anim. Reprod. Sci..

[23] Gonçalves P., Nóbrega L., Monteiro A., Pedreiras P., Rodrigues P., Esteves F. (2021). Sheepit, an E-Shepherd System for Weed Control in Vineyards: Experimental Results and Lessons Learned. Animals.

[24] Ifarmtec Webpage. http://www.ifarmtec.pt.

[25] Tosi J., Taffoni F., Santacatterina M., Sannino R., Formica D. (2017). Performance Evaluation of Bluetooth Low Energy: A Systematic Review. Sensors.

[26] IPMA—Instituto Português do Mar e da Atmosfera. https://www.ipma.pt/pt/produtoseservicos/index.jsp?page=dados.xml.

[27] Yang C.T., Wang C.M., Zhao Y.G., Chen T.B., Aubry A., Gordon A.W., Yan T. (2020). Updating Maintenance Energy Requirement for the Current Sheep Flocks and the Associated Effect of Nutritional and Animal Factors. Animal.

[28] Shinde A.K., Karim S.A. (2007). Energy Expenditure of Sheep and Goats at Pasture—A Review. Indian J. Small Rumin..

